# The Influence of Grit on Life Satisfaction: Self-Esteem as a Mediator

**DOI:** 10.5334/pb.400

**Published:** 2018-04-27

**Authors:** Jie Li, Mengyuan Fang, Wangshuai Wang, Gong Sun, Zhiming Cheng

**Affiliations:** 1 School of Management, Shanghai University, Shanghai, CN; 2 School of Management, Shanghai University of International Business and Economics, Shanghai, CN; 3 Department of Marketing, Central University of Finance and Economics, Beijing, CN; 4 Social Policy Research Centre, The University of New South Wales, New South Wales, AU; 5 Centre for Social Research in Health, The University of New South Wales, New South Wales, AU

**Keywords:** grit, self-esteem, life satisfaction, mediation, China

## Abstract

Improving people’s life satisfaction has become an important goal for many individuals and societies. In this study we investigate how grit influences life satisfaction. We propose that individuals’ self-esteem mediates the relationship between grit and life satisfaction. Study 1, with a sample of 243 employees enrolled in a business training course, found that an individual’s grit was positively related to life satisfaction and that self-esteem fully mediated this relationship. In Study 2, with 218 full-time employees, self-efficacy, self-control, and self-consciousness were included as mediators, but they did not exceed the power of self-esteem in explaining the relationship between grit and life satisfaction. Implications, limitations and future research directions are discussed.

## Introduction

People throughout the world desire better lives in the pursuit of happiness (Cordeiro, Paixão, Lens, Lacante, & Luyckx, 2016). Accordingly, researchers have increasingly focused on what affects individuals’ life satisfaction. There is a widespread idea that individuals’ traits can explain why some are more satisfied with life than others in the same environment. Among factors that include family, friends, finance, and culture (Diener & Diener, 1995; Hofstede, 1980; Triandis, 1989), grit is an important predictor of life satisfaction (Singh & Jha, 2008). In psychology, Duckworth and colleagues (2007) first coined this term to illustrate the personality trait of perseverance and passion for long-term goals. In essence, grit manifests in the decision to complete a long-term goal despite the risk of failure and obstacles in the process (Doskoch & Flora, 2005).

Being more enthusiastic and scrappy, people with a high level of grit go further in the pursuit of their goals and dreams. Therefore, grit predicts individuals’ hardiness and resilience (Vainio & Daukantaite, 2016). In addition, grit interacts with individuals’ potential to predict good grades in school or success in their career (Weisskirch, 2016). Owing to the importance of grit, researchers have investigated its antecedents and outcomes. For example, Duckworth and colleagues (2007) argue that compared to intelligence, academic performance, or appearance, grit is the most reliable predictor of personal success. Weisskirch (2016) suggests that individuals who feel good and use effective strategies tend to cultivate more passion and perseverance in long-term goals, which in turn produce better results. Moreover, researchers have found that the two components of grit, which are consistency of interests and perseverance of efforts, generate success and happiness for individuals (Duckworth & Gross, 2014; Vainio & Daukantaite, 2016; Von Culin, Tsukayama, & Duckworth, 2014).

Although previous studies have accumulated great knowledge, some issues remain underexplored. For example, although prior theoretical research has indicated that individuals’ grit predicts life satisfaction, little research has empirically examined the underlying mechanism between them. In this article, we propose that self-esteem mediates the relationship between grit and life satisfaction. It is widely accepted that high self-esteem is not only a personal expectation but also the central psychological source of many positive behavior and outcomes. That is, self-esteem has profound influences on every aspect of our lives (Branden, 1994). Thus, over the past few decades, the need for high self-esteem has risen from an individual concern to a societal concern such that high self-esteem bears psychological resources (e.g., optimism and personal resilience) that benefit individuals and societies, whereas low self-esteem lies at the root of individual and societal problems (Mäkikangas, Kinnunen, & Feldt, 2004; Symister & Friend, 2003). Indeed, most social problems or crises can be attributed to individuals’ lack of self-love, which strongly overlaps with self-esteem (Baumeister, Campbell, Krueger, & Vohs, 2003).

To fill this research gap, we choose self-esteem as a mediating variable to explore the relationship between grit and life satisfaction. We choose self-esteem because it reflects one’s subjective evaluation of self-worth (Leary & MacDonald, 2003) and thus influences one’s persistence, competence, and enthusiasm in long-term goals. Furthermore, self-esteem shapes one’s attitudes towards and quality of life. In addition, research suggests that self-esteem mediates the effect of personality traits on one’s life satisfaction (Lai, Bond, & Hui, 2007). In this article, we propose that grit predicts one’s life satisfaction and that self-esteem mediates this relationship. Thus, we propose a theoretical framework that explores how grit affects life satisfaction. Subsequently, we present two empirical studies to examine the hypotheses. Finally, implications and limitations are discussed.

### Girt and self-esteem

Self-esteem is the attitude that a person has towards himself or herself. In fact, self-esteem is the definition of self-worth, and it reflects the subjective evaluation of worth as a person (Rosenberg, 1986). High self-esteem stimulates self-potential (Weisskirch, 2016) and enhances the initiative and pleasure. Facing failure, strong self-esteem allows people to persist for longer (Baumeister et al., 2003). Self-esteem may foretell future externalizing problems (Donnellan, Trzesniewski, Robins, Moffitt & Caspi, 2005). According to social bonding theory, low self-esteem weakens individuals’ relationship with society, reduces the consistency of social norms, and increases the crime rate (Hirschi, 1969).

Although studies have not directly demonstrated how an individual’s grit affects self-esteem, we can find some evidence from the literature. Literature on motivation suggests that people with a high level of grit are likely to be enthusiastic about and persistent in pursuing long-term goals (Von Culin et al., 2014). In this process, with a clearer understanding, they are more satisfied with themselves. Meanwhile, researchers also find a strong connection between grit and self-concept. For example, grit might reflect a particularly strong connection with the self, which in turn enhances self-awareness and self-knowledge and thus contributes to one’s self-concept clarity (Gardner, Avolio, Luthans, May, & Walumbwa, 2005). Self-concept refers to what we think about ourselves, and self-esteem refers to our feelings about the positive or negative assessments of ourselves (Smith & Mackie, 2007). Although self-esteem is more emotional than self-concept (i.e., the extent to which self-beliefs are clearly specified), they are closely related with one another (Campbell et al., 1996).

In addition, grit reflects a motivation towards the future, and it may induce a sense of hope that makes individuals feel better and realize their self-worth (Kleiman, Adams, Kashdan, & Riskind, 2013). A person who feels that life is full of meaning will have the same view about himself or herself; that is, he or she may have high self-esteem.

### Self-esteem and life satisfaction

Life satisfaction refers to one’s cognitive process of evaluation of quality of life (Diener, Emmons, Larsen, & Griffin, 1985; Peterson, Park, & Seligman, 2005). It is a cognitive aspect of subjective well-being that reflects one’s perceived happiness and satisfaction. Life satisfaction is the main evaluation criterion of one’s living environment, interpersonal relationships, and so forth. Life satisfaction is a feeling about oneself, about others, and about trivia in life. Thus, life satisfaction reflects one’s quality of life as a whole (Diener & Diener, 1995).

A previous study (McGillivray, Lau, Cummins, & Davey, 2009) suggests that life satisfaction is a multifaceted construct that refers to one’s overall evaluation of life domains such as health, finances, work, self-esteem, and interpersonal relationships. In other words, self-evaluation is critical for evaluating quality of life as a whole (Çivitci & Çivitci, 2009). Zhang and Leung (2002) argue that in addition to demographic characteristics, social relationships, personality, and coping mechanisms, self-esteem is one of the strongest predictors of life satisfaction. In an American sample, self-esteem has a correlation of 0.55 with life satisfaction (Campbell, 1981). In a survey of Chinese parents whose children have autism spectrum disorder, life satisfaction was associated with higher household income and better self-esteem (Lu et al., 2015). Among the various predictors, self-esteem has the strongest impact on adults’ life satisfaction (Campbell, Converse, & Rodgers, 1976).

### The mediating role of self-esteem

Although prior research has examined the relationship between grit and life satisfaction, the intermediary mechanism underlying this process has not been thoroughly explored. Researchers suggest that self-esteem may mediate the relationship between personality traits and life satisfaction (Joshanloo & Afshanri, 2011; Lai et al., 2007). In this article, we propose that self-esteem mediates the relationship between grit and life satisfaction. That is, individuals’ persistence and enthusiasm in the pursuit of long-term goals affect their self-evaluation, which in turn predicts their evaluation of life.

When people persist in their long-term goals, they may perceive themselves as persistent and feel pride in themselves. Thus, their attitude towards themselves will be positive, which in turn enhances their life satisfaction. In contrast, those who cannot adhere to their long-term goals perceive themselves as powerless. Therefore, they do not feel as good as their counterparts who have a high level of grit.

### Additional potential mediators

We propose some additional potential mediators according to previous literature. Grit is essentially the degree of perseverance and enthusiasm in facing particular situations. The ability to regulate emotions, thoughts, and behaviours (self-efficacy) (Schwarzer, 1992) will improve, and an individual will be more confident about his/her own ability to achieve his/her intended results (self-control) (Diamond, 2013). When an individual is preoccupied with him/herself, his/her self-consciousness is higher. Their effects on life satisfaction might come through direct or indirect routes. For example, when an individual can control his/her emotions to minimize or escape bad feelings and promote good ones, life satisfaction can be improved (Hofmann, Luhmann, Fisher, Vohs, & Baumeister, 2014).

### Current study and hypotheses

Our goal of this study is to examine how grit influences life satisfaction and the role of self-esteem in this relationship. According to the facts detailed in the prior paragraphs, we assume that (1) grit is positively related to self-esteem; (2) self-esteem is positively related to life satisfaction; (3) self-esteem mediates the relationship between grit and life satisfaction; and (4) self-esteem mediates the relationship between grit and life satisfaction when self-efficacy, self-control, and self-consciousness are included as additional mediators.

We conducted two surveys to examine these hypotheses. The participants in Study 1 were employees in a training course. To strengthen the robustness and mitigate the sample limitations in Study 1 (such as the sampling method and sample characteristics), Study 2 used random sampling and added several potential variables to further verify the mediating effect of self-esteem. That is, we propose that self-esteem mediates the relationships between grit and life satisfaction, in which self-efficacy, self-control, and self-consciousness are considered as additional mediators.

## Method

### Participants and Procedures

In Study 1, the participants consisted of 243 employees who were enrolled in a business training course in East China. A paper-and-pencil survey was used to collect the data. We distributed 295 questionnaires to these participants. A cover letter assured that their participation was voluntary and anonymous. Our survey took approximately 10 minutes to complete. After excluding those who did not complete the survey or quit during the process, we obtained a final sample of 243 participants. Among those participants, 98 (40.30%) were male, and 145 (59.70%) were female. The majority (79.4%) were 19 to 29 years old, and the majority (66.7%) held an associate degree. Most (90.9%) had monthly income ranging from RMB 1000 to 10000.

In Study 2, the participants were full-time employees from different locations in China. We hired an online survey company to collect the data. Following a random sampling procedure, the company distributed our questionnaire to participants who were employed full-time. Participation was voluntary, and participants received small monetary incentives from the online survey company. The valid sample size is 218, including 124 females and 94 males. Most of the participants (93.6%) were 19 to 69 years old, 91.7% had a graduate or postgraduate degree, and most of them (98.6%) had an monthly income between RMB 1000 and 50000.

### Measures

All questionnaire items were originally developed in English. To ensure accuracy and clarity, we translated the items into Chinese following back-translation procedures (Brislin, Lonner, & Thorndike, 1973). Then, one native English speaker checked the original items and the corresponding back-translated items to ensure that the Chinese versions of these scales were suitable for our research context. In Study 1, all items used seven-point Likert scales (1 = strongly disagree, 7 = strongly agree), whereas in Study 2, all items used five-point Likert scales (1 = strongly disagree, 5 = strongly agree).

**Grit.** We used Duckworth and Quinn’s (2009) 8-item Short Grit Scale to assess participants’ level of grit. This scale includes two dimensions: perseverance of effort (e.g., “Setbacks don’t discourage me”) and consistency of interest (e.g., “My interests change from year to year”, reverse coded). Li and colleagues (2016) have validated the reliability and validity of this scale for Chinese participants. The Cronbach’s alpha coefficient for Study 1 and Study 2 were 0.76 and 0.82, respectively.

**Self-esteem.** We assessed the participants’ self-esteem using the 10-item Rosenberg Self-esteem Scale (Rosenberg, 1965). Sample items include “I feel that I am a person of worth, at least on an equal plane with others” and “I feel that I have a number of good qualities.” Both the reliability and validity of the Rosenberg Self-esteem Scale are at good levels (Kong & You, 2013; Zhao, Kong, & Wang, 2012). The Cronbach’s alpha coefficient for Study 1 and Study 2 were 0.82 and 0.87, respectively.

**Life satisfaction.** To measure the participants’ overall quality of life, we used the 5-item Satisfaction with Life Scale (SWLS) (Diener et al., 1985). One sample item is “I am satisfied with life.” The participants indicated the degree of agreement with each statement using a seven-point Likert scale. An extensive body of research testifies to the good reliability and validity of the SWLS (Kong & You, 2013; Kong, Zhao, & You, 2012). The Cronbach’s alpha coefficient for Study 1 and Study 2 were 0.90 and 0.88, respectively.

**Self-efficacy.** Self-efficacy assesses people’s judgement of their capabilities to organize and execute courses of action required to attain designated types of performances (Bandura, 1986). For testing participants’ self-efficacy, we used a 6-item Self-efficacy Scale (Speier & Frese, 1997). One item is “I judge my abilities to be high.” In Study 2, the Cronbach’s alpha coefficient was 0.83.

**Self-control.** We used the ten-item measure adapted from Tangney, Baumeister and Boone (2004) to evaluate the ability to regulate one’s emotions, thoughts, and behaviour in the presence of temptations and impulses (DeLisi, 2014; Diamond, 2013). The measure consisted of items such as “I refuse things that are bad for me, even if they are fun”. In Study 2, the Cronbach’s alpha coefficient was 0.84.

**Self-consciousness.** Self-consciousness was measured in Study 2 with the 10-item scale from the International Personality Item Pool (IPIP) version of the Self-consciousness Scale (Fenigstein, Scheier, & Buss, 1975). Sample items include “Am afraid that I will do the wrong thing” and “Am not bothered by difficult social situations”. The Cronbach’s alpha coefficient was 0.82.

**Demographic variables.** To minimize the influence of other exogenous variables, we surveyed the respondents’ gender (1 = male; 2 = female), age (in years), education, and income.

### Data analysis strategy

#### Study 1

To examine our hypotheses, we first computed descriptive statistics for the variables. Next, we examined correlations among all variables. Then, we assessed the validity of the measurement models via confirmatory factor analysis (CFA) prior to the examination of the structural models.

Because the original scales of grit and self-esteem consisted of many items, we simplified them with parceling procedures (Kishton & Widaman, 1994). Importantly, item parceling reduces model complexity and the number of parameters estimated without researchers’ having to pay the price of losing information that may contribute to the meaning of a latent variable (Thompson & Melancon, 1996). This treatment resulted in a final measurement model that included four indicators for grit, five indicators for self-esteem, and five items for life satisfaction.

We adopted structural equation modelling (SEM), which can treat complex models, to examine our hypotheses. The goodness-of-fit of the estimated models was evaluated based on the following criteria: a ratio of the chi-squared value to the number of degrees of freedom ratio (χ^2^/*df*) less than 5, a Comparative Fit Index (CFI) greater than 0.90, a Non-normed Fit Index (NNFI) greater than 0.90, and a Root-mean-square Error of Approximation (RMSEA) less than 0.08 (Browne & Cudeck, 1993; Kline, 2011). Because the sensitivity analysis revealed that the effects of all the control variables were non-significant, we do not report the results for controls for parsimony.

#### Study 2

To address the sampling limitations of Study 1, we conducted a second study. The first aim of Study 2 was to strengthen the robustness of the results. The second aim was to further verify the mediating effect of self-esteem by including three mediators.

To examine the multiple-mediator model, we employed nonparametric bootstrapping procedure (5000 replications) to examine our hypotheses using PROCESS in SPSS (Hayes, 2013). Most prior research examines the mediation effects following Baron and Kenny’s (1986) recommended analysis procedures. However, this classical method may be problematic when analyzing a multiple-mediator model because it assumes that the samples are normally and symmetrically distributed (Preacher & Hayes, 2008). In addition, it suffers from low statistical power, especially when the sample size is small or moderate (MacKinnon, Lockwood, Hoffman, West, & Sheets, 2002).

## Results

### Study 1

#### Descriptive statistics and correlations

Table 1 reports the means, standard deviations, and correlations of the variables. Grit is positively related to self-esteem (*r* = 0.56, *p* < 0.01) and life satisfaction (*r* = 0.28, *p* < 0.01). In addition, self-esteem is positively related to life satisfaction (*r* = 0.43, *p* < 0.01). These results provide initial support for Hypothesis 1 and 2.

**Table 1:** Descriptive Statistics and Correlations of All Study Variables in Study 1.

	Mean	*SD*	1	2	3	4	5	6	7

1. Grit	4.41	0.96	–						
2. Self-esteem	4.86	0.97	0.56**	–					
3. Life satisfaction	3.81	1.22	0.28**	0.43**	–				
4. Gender	1.60	0.49	0.11	0.18**	0.14*	–			
5. Age	2.21	0.42	0.09	0.05	0.71	–0.01	–		
6. Education	3.33	0.52	0.10	0.10	0.06	–0.02	–0.11	–	
7. Income	2.65	0.71	0.07	0.12	0.10	–0.13*	0.29**	0.06	–

*Note: N* = 243. Gender: 1 = male, 2 = female. Age: 1 = under 18 years old, 2 = 19 ~ 29 years old, 3 = 30 ~ 39 years old, 4 = over 40 years old. Education: 1 = middle school and below, 2 = high school, 3 = associate degree, 4 = bachelor’s degree, 5 = master’s degree or above. Income: 1 = less than RMB 1000, 2 = RMB 1001 ~ 5000, 3 = RMB 5000 ~ 10000, 4 = RMB 10001 ~ 15000, 5 = more than RMB 15000. * *p* < .05. ** *p* < .01.

#### Measurement model

According to the two-step approach (Anderson & Gerbing, 1988; Li, Zhang, & Sun, 2015), we used a series of CFAs to assess the properties of a three-factor measurement model (grit, self-esteem, and life satisfaction) via the package “lavaan” (Rosseel, 2012) in R.

We compared the baseline model (M_1_) with five alternative models: one null model (M_0_), two models designed to test whether grit could be distinguished from self-esteem (M_2_) and life satisfaction (M_3_), one model testing whether self-esteem could be distinguished from life satisfaction (M_4_), and one model suggesting that three variables represent a single indicator (M_5_). The proposed three-factor baseline model provided the best fit index (χ^2^ [74] = 201.82, *p* < 0.001; CFI = 0.94, NNFI = 0.92, RMSEA = 0.08) relative to the other alternative models. Therefore, the three constructs can be distinguished well. Detailed results are presented in Table 2.

**Table 2:** Confirmatory Factor Analyses of Measurement Models in Study 1.

Model Specifications	*χ*^2^	*df*	*Δχ*^2^	CFI	NNFI	RMSEA

Null model (M_0_)	2102.65	91	–	–	–	–
Baseline 3-factor model (M_1_)	201.82	74	–	0.94	0.92	0.08
Grit and self-esteem combined (M_2_)	380.47	76	178.65**	0.85	0.82	0.13
Grit and life satisfaction combined (M_3_)	595.24	76	214.77**	0.74	0.69	0.17
Self-esteem and life satisfaction combined (M_4_)	615.41	76	20.17**	0.73	0.68	0.17
Three constructs represent a single dimension (M_5_)	931.50	77	316.09**	0.58	0.50	0.21

*Notes: N* = 243. *Δχ*^2^ is the change in *χ*^2^ compared with the baseline model. * *p* < .05. ** *p* < .01.

#### Structural model

We compared a series of structural models to test all the hypotheses (Table 3). The baseline model, Model 1, is a full mediation model including a path from grit to self-esteem and a path from self-esteem to life satisfaction. Model 2 is a partial mediation model with the addition of a direct path from grit to life satisfaction. Self-esteem is a common cause of both grit and life satisfaction in Model 3. The common cause in Model 4 is grit. Finally, Model 5 is still a full mediation model from self-esteem to grit and grit to life satisfaction.

**Table 3:** Comparisons of Structural Equation Models in Study 1.

Model Specifications	*χ*^2^	*df*	*BIC*	CFI	NNFI	RMSEA

1. Grit→self-esteem→life satisfaction^a^	201.98	75	12588.69	0.94	0.92	0.08
2. Grit→self-esteem→life satisfaction & Grit→life satisfaction	201.82	74	12594.02	0.94	0.92	0.08
3. Self-esteem→grit & Self-esteem→life satisfaction	201.82	74	12594.02	0.94	0.92	0.08
4. Grit→self-esteem & Grit→life satisfaction	201.82	74	12594.02	0.94	0.92	0.08
5. Self-esteem→grit→life satisfaction	232.28	75	12618.99	0.92	0.91	0.09

*Notes: N* = 243. *Δχ*^2^ is the change in *χ*^2^ compared with the baseline model. * *p* < .05. ** *p* < .01. ^a^ Baseline model.

According to these principles illustrated in the “Data analysis strategy” section, the baseline model (Model 1) yielded the best fit to the data (*χ^2^* [75] = 201.98, *p* < 0.001; BIC = 12588.69, CFI = 0.94, NNFI = 0.92, RMSEA = 0.08). Although the alternative models exhibited similar fits (the changes in *χ*^2^ were insignificant), we selected Model 1 based on parsimony. Because both Model 1 and Model 2 were not nested, we compared their Bayesian Information Criterion (BIC) values. The results (Model 1 BIC = 12588.69, Model 2 BIC = 12594.02) indicate that Model 1 fit the data better. In conclusion, self-esteem fully mediated the effects of grit and life satisfaction.

**Figure 1:**
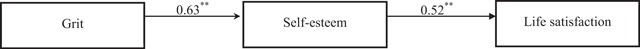
Path Coefficients of the Hypothesized Model. *Notes: N* = 243. * *p* < .05. ** *p* < .01.

Figure 1 demonstrates the path coefficients of the final selected model. The paths from both grit to self-esteem (β = 0.63, *p* < 0.01) and self-esteem to life satisfaction (β = 0.56, *p* < 0.01) are significant, in support of Hypotheses 1 and 2. The mediating effect of self-esteem proposed in Hypothesis 3 is also supported.

To further test Hypothesis 3, we adopted boot-strapping procedures (1000 replications) to examine the mediating role of self-esteem in the hypothesized model. The boot-strapping approach enhances the statistical power of mediation analysis, especially when the sample size is small or moderate (Preacher & Hayes, 2008). Table 4 demonstrates the direct effects and their associated 95% confidence intervals. The direct effects of grit on self-esteem and self-esteem on life satisfaction are both significant. Moreover, the indirect effect of grit on life satisfaction through self-esteem (γ = 0.33; *p* < 0.01; 95% CI [0.24, 0.42]) is significant. Therefore, all the hypotheses were supported.

**Table 4:** Parameter Estimates of the Mediation Model and 95% Confidence Intervals in Study 1.

	Estimated effect	95% CI^a^

**Direct effects**		
grit→self-esteem	0.63**	[0.53, 0.72]
self-esteem→life satisfaction	0.52**	[0.42, 0.63]
**Indirect effect**		
grit→self-esteem→life satisfaction	0.33**	[0.24, 0.42]

*Notes: N* = 243. ^a^ CI = confidence interval (1000 bootstrap samples). * *p* < .05. ** *p* < .01.

#### Gender Differences

The correlation analysis reveals no gender differences in grit, while female participants have higher self-esteem and life satisfaction. To validate our results, we compared the path coefficients across genders using multi-group analysis. The results show non-significant chi-square differences between two models (Δχ^2^ = 18.59, *df* = 13, *p* = 0.14). Moreover, inspection of path coefficients confirms that all the paths do not differ between genders.

#### Age Differences

In this study, we measured the participants’ ages using a categorical variable. None of them was under 18 years old and only one participant was over 40 years old. Therefore, we divided our participants into two groups (under 29 years, *N* = 193; over 30 years old, *N* = 50). We conducted multi-group analysis to examine the differences in structural models across age groups. The results show non-significant chi-square differences between two models (*Δχ*^2^ = 11.00, *df* = 13, *p* = 0.61) Moreover, inspection of the path coefficients confirms that all the paths do not differ across age groups.

### Study 2

#### Descriptive statistics and correlations

Study 1 found that self-esteem serves as a mediator in the relationship between grit and life satisfaction. In Study 2, self-efficacy, self-control, and self-consciousness were included as mediators. We will confirm the role of self-esteem in explaining the relationship between grit and life satisfaction.

Table 5 presents the descriptive statistics and correlations among grit, self-esteem, self-efficacy, self-control, self-consciousness and life satisfaction. Grit was positively related to self-esteem (*r* = 0.67, *p* < 0.01), self-efficacy (*r* = 0.65, *p* < 0.01), self-control (*r* = 0.71, *p* < 0.01) and life satisfaction (*r* = 0.47, *p* < 0.01), and negatively related to self-consciousness (*r* = –0.64, *p* < 0.01). Additionally, the correlations between life satisfaction and self-esteem (*r* = 0.65, *p* < 0.01), self-efficacy (*r* = 0.55, *p* < 0.01), self-control (*r* = 0.51, *p* < 0.01) were positive, whereas the correlation between self-consciousness and life satisfaction was negative (*r* = –0.56, *p* < 0.01).

**Table 5:** Descriptive Statistics and Correlations of All Study Variables in Study 2.

	Mean	*SD*	1	2	3	4	5	6	7	8	9	10

1. Grit	3.80	0.60	–									
2. Self-esteem	3.86	0.56	0.67**	–								
3. Self-efficacy	3.97	0.59	0.65**	0.69**	–							
4. Self-control	3.41	0.64	0.71**	0.60**	0.57**	–						
5. Self-consciousness	2.35	0.59	–0.64**	–0.66**	–0.63**	–0.68**	–					
6. Life satisfaction	3.45	0.79	0.47**	0.65**	0.55**	0.51**	–0.56**	–				
7. Gender	1.57	0.50	–0.03	0.07	–0.02	0.02	–0.03	0.04	–			
8. Age	3.70	0.98	0.09	0.04	0.14*	0.06	–0.02	0.05	–0.08	–		
9. Education	3.00	0.40	0.06	0.11	0.11	–0.05	–0.07	0.04	0.11	–0.02	–	
10. Income	3.89	0.78	0.20**	0.19**	0.19**	0.15*	–0.24**	0.17*	–0.21**	0.11	0.22**	–

*Note: N* = 218. Gender: 1 = male, 2 = female. Age: 1 = under 18 years old, 2 = 19 ~ 29 years old, 3 = 30 ~ 39 years old, 4 = 40 ~ 49 years old, 5 = 50 ~ 59 years old, 6 = 60 ~ 69 years old, 7 = over 70 years old. Education: 1 = middle school and below, 2 = high school, 3 = associate degree, 4 = bachelor’s degree, 5 = master’s degree or above. Income: 1 = less than RMB 1000, 2 = RMB 1001 ~ 3000, 3 = RMB 3001 ~ 5000, 4 = RMB 5001 ~ 10000, 5 = RMB 10001 ~ 50000, 6 = more than RMB 50000. * *p* < .05. ** *p* < .01.

#### Hypothesis testing

**Figure 2:**
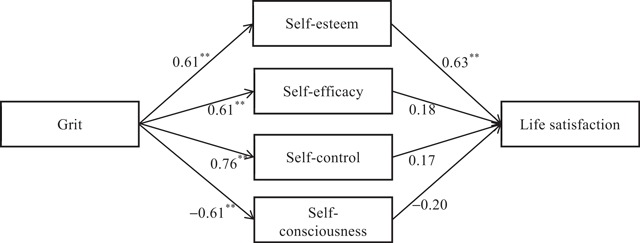
Results of Multiple-Mediator Model in Study 2. *Notes: N* = 218. * *p* < .05. ** *p* < .01.

The standardized path estimates in Figure 2 indicate support for Hypotheses 1 and 2 because the paths from grit to self-esteem (β = 0.61, *p* < 0.01) and self-esteem to life satisfaction (β = 0.63, *p* < 0.01) were positive and statistically significant. In other words, people who have higher level girt tend to have high self-esteem and be more satisfied with life. Hypothesis 3 was also supported.

The results presented in Table 6 indicate that for life satisfaction as a dependent variable, after control variables were taken into account, grit is positively related to self-esteem (β = 0.61; *p* < .01), self-efficacy (β = 0.61; *p* < .01) and self-control (β = 0.76; *p* < .01) and negatively related to self-consciousness (β = –0.61; *p* < .01). When controlling for grit, only self-esteem is positively related to life satisfaction (β = 0.63; *p* < .01). Further results indicate that the indirect effect of grit on life satisfaction through self-esteem (γ = 0.38; *p* < .01; 95% CI [0.21, 0.60]) is uniquely significant. Because the direct effect is non-significant, the effect of grit on life satisfaction is fully mediated by self-esteem. Taken together, the mediating roles are confirmed, supporting Hypothesis 4.

**Table 6:** Results of Multiple Mediation Analyses in Study 2.

	Mediators							
	Self-esteem		Self-efficacy		Self-control		Self-consciousness	

Path analysis	β	*SE*	β	*SE*	β	*SE*	β	*SE*

IV-M	0.61**	0.05	0.61**	0.05	0.76**	0.05	–0.61**	0.05
M-DV	0.63**	0.11	0.18	0.10	0.17	0.10	–0.20	0.11
**Bootstrapping**								

Indirect effect through M	0.38**	0.10	0.11	0.07	0.13	0.08	0.12	0.08
BC 95% CI^a^	0.21	0.60	–0.03	0.25	–0.02	0.29	–0.03	0.27
**Effect of IV on DV**	β	*SE*						

Total effect	0.74**	0.10						
Direct effect	–0.14	0.11						

*Note: N* = 218. *p < .05; **p < .01. IV = grit; DV = life satisfaction. ^a^BC = bias-corrected; CI = confidence interval (5000 bootstrap samples). Owing to space limitations, the results for control variables are not reported here but are available from the authors.

## Discussion

According to organismic valuing theory, we infer that high grit leads to high self-esteem and high life satisfaction. Specifically, we investigated the mediating role of self-esteem in the relationship between grit and life satisfaction. We conducted two questionnaire surveys to examine our hypotheses. In Study 1, the results indicated that self-esteem mediates the relationship between grit and life satisfaction. To further verify the mediating effect, we added three potential mediators in Study 2. A bootstrapping approach was adopted to test the significance of the mediating role of self-esteem, self-efficacy, self-control, and self-consciousness in the hypothesized model. The results reveal that only self-esteem mediates the relationship between grit and life satisfaction. In conclusion, our findings indicate that individuals with more passion and perseverance in long-term goals possess more positive attitudes towards themselves, which has a strong connection to the evaluation of quality of life.

### Theoretical and Practical Implications

This article contributes to the literature in several ways. First, it extends the research on life satisfaction and finds that grit plays a positive role in affecting life satisfaction. In contrast to the prior studies that merely reported a positive correlation between grit and life satisfaction (Singh & Jha, 2008), our results indicate that grit influences life satisfaction. Second, comparing extraneous factors (such as income), this study explores a new branch in the field of the psychosocial factors affecting life satisfaction. Third, this work provides a better understanding of the etiology of life satisfaction. Apart from the vastly influenced external conditions, traits that have been formed for a long time will determine the standard of judgement, further affecting one’s feelings toward and evaluation of life.

Our findings also have several implications for practice. Individuals’ satisfaction with life is of great significance for both people and society. Studies indicate that low life satisfaction predicts mortality and high suicidality (Lightsey, Maxwell, Nash, Rarey & McKinney, 2011). People who are more satisfied with life tend to be more positive, healthier, and happier. In addition, society is more harmonious when people are content with life. How to improve life satisfaction has been a longstanding topic for both individuals and society. Our research suggests that grit and self-esteem may influence one’s life satisfaction in both direct and indirect manners. Thus, we can attempt to use certain methods to improve the level of grit or self-esteem. First, this can provide some insight about education. Both families and schools have the duty to encourage children to do what they love longer and teach them how to adapt themselves in a correct manner. Second, due to the connection between life satisfaction and suicidality (Lightsey et al., 2011), we should pay attention to offenders’ self-esteem construction. Third, we need to attempt to help individuals with low self-esteem, especially those at the bottom of society, by providing both material assistance and respect.

### Limitations and Directions for Future Research

Several limitations of the current study should be addressed. First, the data were collected from adults in China. Although our sampling may exclude potential influences of other variables, such as participants’ living environment and social status, the homogeneity of the sample limits the extent to which these findings can be generalized to other countries. Second, the survey was completed through self-report measures, which may be a threat to the objectivity of our data. This concern is alleviated by the fact that all the study variables are individuals’ perceptions or psychological states. Thus, it is unreasonable for others to observe an individual’s grit, self-esteem, and life satisfaction. Third, this study was a cross-sectional design. Thus, we could not determine a causal relationship based on the results. Longitudinal or experimental studies should be conducted in the future when considering the reciprocal associations among grit, self-esteem, and life satisfaction (Shadish, Cook, & Campbell, 2002). Finally, we need future studies to repeatedly examine the validity of the Chinese versions of scales, although we have tested them.

Future research should explore whether there are common causes, such as temperament or broader personality traits, that may lead to high levels of grit and life satisfaction. Furthermore, we could adopt a longitudinal or experimental design to confirm causal relationships between grit, self-esteem and life satisfaction. We could also use multiple methods for evaluation, such as evaluations by parents and friends. In addition, the Chinese version of the scales must be further examined to confirm the validity of the current research.
